# Carob Extract (*Ceratonia siliqua* L.): Effects on Dyslipidemia and Obesity in a High-Fat Diet-Fed Rat Model

**DOI:** 10.3390/pharmaceutics15112611

**Published:** 2023-11-10

**Authors:** Aleksandar Rašković, Nikola Martić, Ana Tomas, Bojana Andrejić-Višnjić, Milana Bosanac, Marko Atanasković, Marko Nemet, Radmila Popović, Marko Krstić, Saša Vukmirović, Nebojša Stilinović

**Affiliations:** 1 Department of Pharmacology, Toxicology, and Clinical Pharmacology, Faculty of Medicine, University of Novi Sad, 21000 Novi Sad, Serbia; aleksandar.raskovic@mf.uns.ac.rs (A.R.); ana.tomas@mf.uns.ac.rs (A.T.); sasa.vukmirovic@mf.uns.ac.rs (S.V.); nebojsa.stilinovic@mf.uns.ac.rs (N.S.); 2 Department of Histology and Embryology, Faculty of Medicine, University of Novi Sad, 21000 Novi Sad, Serbia; bojana.andrejic-visnjic@mf.uns.ac.rs (B.A.-V.); milana.bosanac@mf.uns.ac.rs (M.B.); 3 Faculty of Medicine, University of Novi Sad, 21000 Novi Sad, Serbia; atanaskovic.marko5@gmail.com (M.A.); markonemet@uns.ac.rs (M.N.); radmila.popovic@mf.uns.ac.rs (R.P.); 4 Clinical Department for Anesthesia, Intensive Care and Pain Management, Clinical Centre of Vojvodina, 21000 Novi Sad, Serbia; 5 Faculty of Chemistry, University of Belgrade, 11000 Belgrade, Serbia; mkrstic109@gmail.com

**Keywords:** carob extract, dyslipidemia, obesity, high-fat diet-fed rat model, adiponectin, leptin, CYP2E1, Iba1

## Abstract

Dyslipidemia and obesity are recognized as two of the major global health issues and main risk factors for coronary heart disease and cerebrovascular disease. In recent years, carob has shown certain antioxidant and anti-dyslipidemic potential. In this study, Wistar rats were fed with a standard and cholesterol-enriched diet and treated orally with carob extract and simvastatin for four weeks. After sacrifice, blood samples were collected for biochemical analysis, and liver tissue was taken for histological and immunohistochemical assessment. Weight gain was significantly higher in groups fed with cholesterol-fortified granules; total cholesterol was found to be significantly lower in the hypercholesterolemic groups treated with simvastatin and simvastatin/carob combined regimens compared with hypercholesterolemic animals treated with saline (*p* < 0.05). The same was true for low-density lipoprotein cholesterol and the LDL/HDL ratio (*p* < 0.05). Adiponectin was remarkably higher in animals treated with simvastatin compared to all other groups (*p* < 0.05). Leptin was significantly lower in groups treated with carob and simvastatin compared to the hypercholesterolemic group treated with saline (*p* < 0.05). Carob/simvastatin co-administration reduced hepatocyte damage and improved liver morphology. A study confirmed the anti-dyslipidemic, anti-obesity, and hepatoprotective potential of carob pulp alone or in combination with simvastatin in the treatment of high-fat diet-fed rats.

## 1. Introduction

Dyslipidemia and obesity, two of the main risk factors for coronary heart disease (CHD) and cerebrovascular morbidity and mortality, have been recognized as an ever-increasing global health issue [1,2]. The majority of obese patients are dyslipidemic and have other metabolic changes [3,4]. Dyslipidemia is defined as a disparity in lipid levels: elevated levels of total cholesterol (TC), low-density lipoprotein cholesterol (LDL-C), and triglycerides (TG), and decreased levels of high-density lipoprotein cholesterol (HDL-C) [5]. Regardless of its cause, dyslipidemia initiates the atherogenic process, increasing the risk of cardiovascular diseases [6]. Globally, dyslipidemia accounts for over 50% of coronary heart disease (CHD) cases and contributes to around four million deaths annually, as reported by the World Health Organization [7].

The management of dyslipidemia and obesity is of utmost importance. Current pharmacological therapy regimens for dyslipidemia typically involve statin-based therapy as the first-line approach [8]. Combined with lifestyle modifications and a proper diet, which are the backbone of obesity prevention and treatment, this strategy has shown significant efficacy in treating dyslipidemia and obesity as well [3]. However, long-term use of lipid-lowering agents is not without risks [9]. Diverse side effects of lipid-lowering medications have been well recognized, especially when increasing dosage or combining different agents. The adverse effects of the highest concern are myopathy, neuropathy, liver failure, mental status alterations, and an increased risk of diabetes [9]. Along with the economic burden, these side effects contribute to low adherence to standard treatment [10]. Therefore, there is a constant demand for better alternative or adjunct therapeutic options. Discovering agents with fewer side effects and a lower cost would be reasonable [11].

In recent years, carob (*Ceratonia siliqua* L.) has emerged as a novel potential lipid-lowering plant-based remedy. Carob is an evergreen tree or shrub from the *Caesalpinioideae* sub-family of the *Fabaceae* (*Leguminosae*) family, originally from the Mediterranean region and the Middle East, and it shows certain potential in the food industry [12,13,14]. With Portugal, Italy, and Morocco being major carob cultivators, it is now known worldwide for its edible fruit pods [12]. Over time, carob has gained recognition for its multiple health benefits, mainly anti-inflammatory, anti-oxidant, anti-obesity, and lipid-lowering effects in animals and humans [15]. A series of recent animal and human studies have investigated the effect of carob extracts on the lipid profile [16,17,18,19,20,21,22,23]. Animal studies consistently demonstrate that carob supplementation significantly reduces TC, LDL-C, and TG levels [16,17,18,19,24,25,26,27,28]. Moreover, several studies have shown increased levels of HDL-C after carob consumption [16,17,18,19,29]. Additionally, some studies have reported decreased levels of very low-density lipoprotein cholesterol (VLDL-C) [16,17,18,19].

Insoluble fiber and polyphenols are the two main active ingredients of carob, responsible for its lipid-lowering effects [15]. These components work synergistically through a complex mechanism of action, but their target organ and mechanism of action are different. Insoluble fibers could affect the gastrointestinal tract and, in that way, lower lipid levels. On the other side, polyphenols have some effects on hepatic metabolism pathways through enzyme induction or inhibition [15].

Secondly, carob increases adiponectin (ApN) concentrations [27], leading to decreased serum lipid levels. This occurs through ApN-induced inhibition of lipogenesis in hepatocytes and acceleration of β-oxidation and fatty acid transport in myocytes [30]. These mechanisms not only exert hypolipidemic effects but also may contribute to the anti-obesity effect [31].

Thirdly, carob has been found to decrease levels of leptin [32], a hormone produced by adipocytes that regulates food intake and body weight control. Obese individuals often have high leptin levels due to leptin resistance [33].

These highly beneficial health characteristics of carob come along with only a few rare side effects reported by workers exposed to carob, which include urticaria, asthma, and rhinitis, all attributed to the hypersensitivity of these individuals [34]. No other adverse effects of carob have been reported to date. Regarding herb-to-drug interactions, it was reported that carob bean gum should be administered separately from other oral agents for several hours due to the shortening bowel time effect of carob’s insoluble fibers [34]. No additional interactions have been reported.

To our knowledge, no study has investigated the effectiveness of carob as an adjunct treatment to conventional statin therapy for dyslipidemia and obesity. Our objective was to evaluate the lipid-lowering and anti-obesity effects of carob extracts when administered alone and in combination with simvastatin. We compared these effects to statin monotherapy in dyslipidemic Wistar rats. Further, we aimed to assess the safety of the carob and monitor for potential interactions between the herb and the drug.

## 2. Materials and Methods

### 2.1. Materials

Carob (*Ceratonia siliqua* L., sub-family *Caesalpinioideae*, family *Fabaceae*) extract was prepared from carob pulp flour, a commercial product cultivated in Croatia and processed (deseeded, ground, and roasted), packed, and sold by Aroma začini, D.O.O. (commercially available in Serbia). The preparation of the optimized carob extract (*Ceratonia siliqua* L., sub-family *Caesalpinioideae*, family *Fabaceae),* its chemical characterization, and its antioxidant profile were previously described [35]. Briefly, the extraction procedure was based on an optimal microwave-assisted extraction process. Parameters for maximizing total antioxidant activity were determined by response surface methodology with the following conditions—ethanol concentration 40%, time 25 min, and power 800 W. The obtained ethanol carob extract was evaporated to dryness, then dissolved in distilled water and stored at 4 °C. The extract was in the form of a suspension, and before being administered to the animals, the extract was warmed to room temperature and then stored again at 4 °C before next use. Standard rat pellet food, simvastatin, cholesterol, and cholic acid were purchased from Acros Organics, Geel, Belgium. Rat adiponectin (ab239421) and leptin (ab100773) ELISA kits were purchased from Abcam. The antibodies used for immunohistochemical staining were CYP2E1 (1:200, CSB-PA006425EA01H4, Flarebio, College Park, MD, USA) and Iba-1 (1:8000, AB178847, Abcam, Cambridge, UK). All other chemicals used were of analytical grade.

### 2.2. Experimental Animals and Ethics Clearance

The animals for this experiment were healthy adult male laboratory Wistar albino rats, weighted 225–275 g. They were procured from the Military Medical Academy (Belgrade, Serbia), by whom they were randomly selected. During the entire experiment, rats were housed in the vivarium of the Department of Pharmacology, Toxicology, and Clinical Pharmacology of the Faculty of Medicine in Novi Sad. They were under the standard laboratory conditions: temperature between 23 and 25 °C, humidity of 55 ± 1.5%, a 12 h dark-night cycle, and free, unlimited, all-time access to water and pellet food. The approval for this study was obtained by the Ethics Committee for the Protection of Laboratory Animal Welfare of the University of Novi Sad (Novi Sad, Serbia; No. 04-81/103) and the Ministry of Agriculture and Environmental Protection of the Republic of Serbia (Belgrade, Serbia; No. 323-07-10504/2020-05).

### 2.3. Experimental Design

A total of 40 animals were randomly divided into five different groups of 8 rats. The first group (Group I) was set to be the (non-hypercholesterolemic) control group, which included rats fed with standard pellet food only and provided with a standard water supply. The other four groups (Groups II-V) were intended to represent the hypercholesterolemic groups. For the induction of hypercholesterolemia, rats were fed pellet food fortified with 3% cholesterol and 0.5% cholic acid per kg and supplied with 10% glucose-fortified water for four weeks. After the hypercholesterolemia was induced, the experimental phase began. All five groups differed in terms of the treatment received, as shown in the table below (Table 1).

During the treatment phase, animals were continuously fed with the standard pellet formula (Group I) or the cholesterol- and cholic acid-enriched pellet formula (Group II-V). Both food and water intakes were monitored. The carob extract dose of 400 mg/kg BW was chosen from the available literature and after our pilot study, in which this dose was found to be a safe and optimal dose in rats. The dose of simvastatin for animals was calculated according to the recommended daily dose for human adults weighing 75 kg by using a formula according to the FDA “Guidance for Industry.” Simvastatin was dissolved in saline for 30 min before administration and then given to the animals in the form of an oral solution by an orogastric tube. The body weight of the animals was measured at the beginning and end of the experiment. Ultimately, the animals were inducted into general anesthesia induced by urethane (0.75 g/kg, intraperitone-ally), and then sacrificed by cardiac puncture. Blood samples and tissue specimens were collected for further laboratory analysis, histopathology, and immunohistochemistry assessments.

### 2.4. Lipid Profile, Liver Function Tests, Renal Function Tests, and Adipose-Derived Hormones

The animals’ blood samples were used to determine the lipid status, liver function tests (LFT), renal function tests (RFT), and adipose tissue hormones. The lipid profile was analyzed by applying standard spectrophotometric methods to commercially accessible kits. The levels of TC, LDL-C, HDL-D, VLDL-C, and TG were obtained. To investigate the potential side effects of carob alone and in combination with simvastatin, the three main serum enzymes indicating liver function were examined: alanine transaminase (ALT), aspartate transaminase (AST), and alkaline phosphatase (ALP). The liver function was also explored by probing the levels of total and direct bilirubin. The kidney function was assessed by recording the serum creatinine (sCr), urea (UR), and uric acid (UA) concentrations. All laboratory analyses were performed on an Abbot Alinity Analyzer (Chicago, IL, USA) using commercially available kits. Finally, intending to evaluate the hypothesized mechanisms of action of carob extract on lipid profile and body weight, the serum concentrations of the two major adipose tissue hormones—adiponectin and leptin—were measured using commercial rat ELISA kits, per manufacturer instructions.

### 2.5. Histopathology and Immunohistochemistry Assessment

Two researchers executed the blinded histopathologic analysis of each animal’s liver tissue specimen by using a light microscope. Liver tissue samples were fixed in Bouin’s fixative for 24 h. Thereafter, tissue dehydration with isopropyl alcohol and embedding in liquid paraffin were performed. Using a rotation microtome (Sakura Finetek USA, Inc., Torrance, CA, USA), four successive 5 µm-thick tissue sections were sliced for each rat. Subsequently, two sections were used for the standard hematoxylin and eosin (HE) and Periodic Acid Schiff (PAS) staining, while the other two sections were subjected to immunohistochemical staining with CYP2E1 (1:200, CSB-PA006425EA01H4, Flarebio, College Park, MD, USA) and Iba-1 ((1:8000, AB178847, Abcam, Cambridge, UK)) antibodies, according to the manufacturer’s instructions. All stained sections were observed under a light microscope (BX-43, Olympus, Tokyo, Japan) and photographed with an Olympus DP-73 video camera (Olympus, Tokyo, Japan), objectives 20× and 40× were used. The immunohistochemical staining was performed in accordance with the manufacturers’ instructions. The Image J program (version 1.51h, National Institute of Health, Bethesda, MD, USA, freely available at https://imagej.nih.gov/ij/download.html (accessed on 23 March 2023)) was applied for morphometric analyses of the slides stained by CYP2E1 and Iba-1. CYP2E1 staining discerned the cells with cytochrome P450 2E1 isoenzyme activity, whereas the Iba-1 antibody marked liver macrophages. Using 5 high-power field (HPF) photographs, via Image J software, we determined the percentage with CYP2E1 positivity. Also, the mean ratio of Iba-1-positive (Iba1+) cells on 5 HPF (objective 40×) photography was calculated based on the total number of cells in the slide and the number of Iba1+ positive cells.

### 2.6. Statistical Analysis

All the data were analyzed using Origin 2018 software (OriginLab Corp., Northampton, MA, USA). The comparison between five different animal groups was performed using a one-way analysis of variance (ANOVA), followed by Tukey’s post hoc test. The body weights of the animals were compared by a paired, two-tailed Student’s t-test. The data are shown as the mean ± standard deviation (SD).

## 3. Results

### 3.1. Animal Body Mass and Liver Mass Measurement, Food, and Water Intake

Table 2 shows differences between the five groups according to their initial and final body weight, weight gain, liver weight, and food and water intake.

Weight gain was significantly higher in groups II, IV, and V compared to group I (*p* < 0.05). Furthermore, the hypercholesterolemic group treated with carob had remarkably lower weight gain compared to the hypercholesterolemic animal treated with saline (*p* < 0.05). Moreover, groups treated with carob also had notably lower body weight gain than groups treated with simvastatin or simvastatin/carob combined (*p* < 0.05).

Postmortem liver weight was significantly higher in all groups compared to the animals fed t standard pellet food and treated with saline (*p* < 0.05), while it was significantly lower in groups treated with carob, simvastatin, or simvastatin/carob combined compared to hypercholesterolemic animals treated with saline (*p* < 0.05).

Food intake was higher in the group of animals fed with standard food in comparison with other groups (*p* < 0.05). The same was true for water intake, but water intake was also higher in the hypercholesterolemic group treated with saline and in the hypercholesterolemic groups treated with simvastatin or simvastatin/carob combined, in contrast to the hypercholesterolemic group treated with carob (*p* < 0.05).

### 3.2. Effects of Carob Extract on Lipid Profile

The comparisons of lipid profiles between groups are shown in Table 3.

Compared with group II, total cholesterol was found to be significantly lower in the hypercholesterolemic group treated with simvastatin alone or a simvastatin/carob combined regimen (*p* < 0.05). The same was true for LDL-C and the LDL/HDL ratio (*p* < 0.05). No other significant differences in TC, LDL-C, and LDL/HDL ratio were found between groups, except that Group I had lower levels of those in contrast to hypercholesterolemic groups (*p* < 0.05). In terms of HDL-C, a significant difference was found between Group I and Group II, where HDL-C was, as expected, higher in non-hypercholesterolemic groups (*p* < 0.05). More importantly, remarkably higher HDL-C levels were also found in the hypercholesterolemic group treated with the combination of simvastatin and carob compared to the hypercholesterolemic group treated with saline (*p* < 0.05). Regarding TG, there was no notable distinction between groups.

### 3.3. Effects of Carob Extract on Adipocyte-Produced Hormones

**Figure 1:**
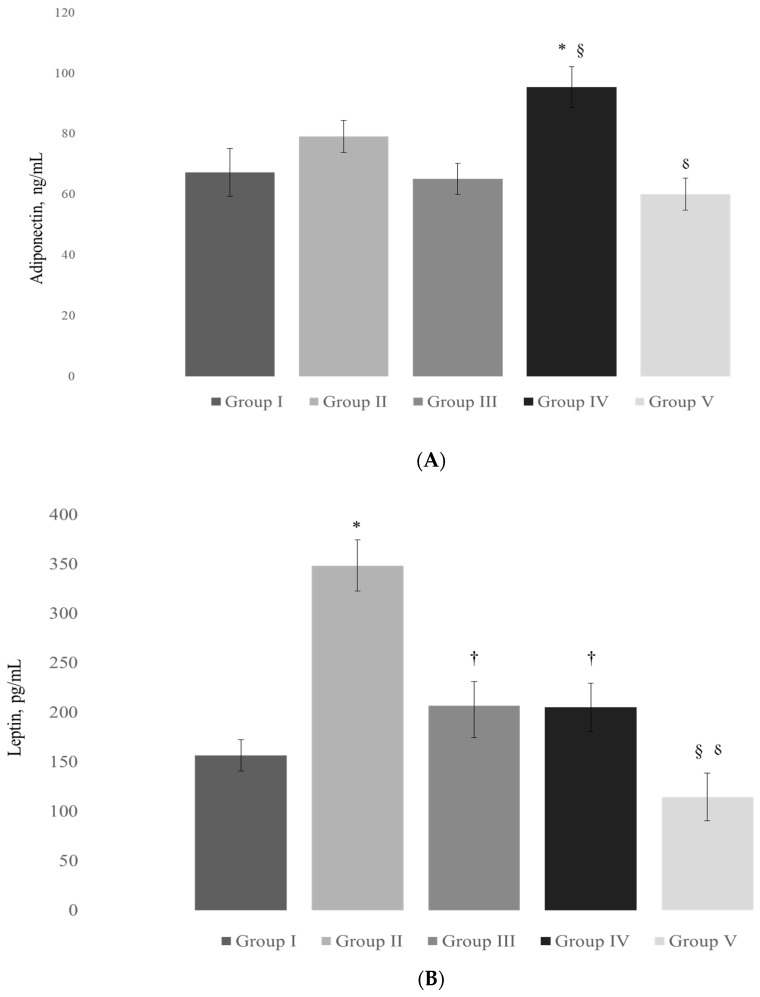
Adiponectin (**A**) and leptin (**B**) levels according to the animal groups. Legend: Data are presented as mean ± SD; * *p* < 0.05 comparing to Group I; ^†^ *p* < 0.05 comparing to Group II; ^§^ *p* < 0.05 comparing to Group III; ^⸹^ *p* < 0.05 comparing to Group IV. Group I—animals treated with saline (1 mL/kg); Group II—hypercholesterolemic animals treated with saline (1 mL/kg); Group III—hypercholesterolemic animals treated with carob extract (400 mg/kg); Group IV—hypercholesterolemic animals treated with simvastatin (10 mg/kg); Group V—hypercholesterolemic animals treated with the combination of carob extract (400 mg/kg) and simvastatin (10 mg/kg).

The comparisons between the levels of adipocyte-produced hormones between groups are shown in Figure 1 (A, adiponectin; B, leptin).

Adiponectin was remarkably higher in animals treated with simvastatin compared to all other groups (*p* < 0.05). Leptin, on the other hand, was significantly lower in groups treated with carob and/or simvastatin compared to the hypercholesterolemic group treated with saline (*p* < 0.05), which had significantly higher leptin levels compared to the control group (*p* < 0.05). Also, levels of leptin were lower in the group treated with the combination of carob and simvastatin compared to the carob-only or simvastatin-only regimen (*p* < 0.05).

### 3.4. Effects of Carob Extract on Serum Biochemical Parameters

Data regarding liver function tests and renal function tests according to the specific animal group are presented in Table 4.

The hypercholesterolemic group treated with carob had remarkably lower ALT levels in comparison to healthy animals treated with saline, the hypercholesterolemic group treated with saline, and the hypercholesterolemic group treated with simvastatin only (*p* < 0.05). There was no significant difference in the renal function tests between hypercholesterolemic groups.

### 3.5. Histological, Immunohistochemical and Morphometric Analysis of Liver Tissue

The liver tissue of Group II animals, as opposed to Group I, showed impaired cellular morphology. Most of the hepatocytes in each liver lobule revealed the accumulation of micro- and macro-vesicular intracellular lipid droplets (steatosis). In addition to steatosis, hepatocyte ballooning was present, along with rare inflammatory foci composed of mononuclear inflammatory cells. Necrosis and fibrosis were not found. Carob/simvastatin co-administration (Group V) reduced hepatocyte damage and improved liver morphology, but carob alone (Group III) nearly annulled steatosis and ballooning. Simvastatin alone administration alleviated hepatic tissue impairment, but to a lesser extent compared to carob or carob/statin administration (steatosis is present but less compared to Group II; no inflammatory cells were found).

**Figure 2:**
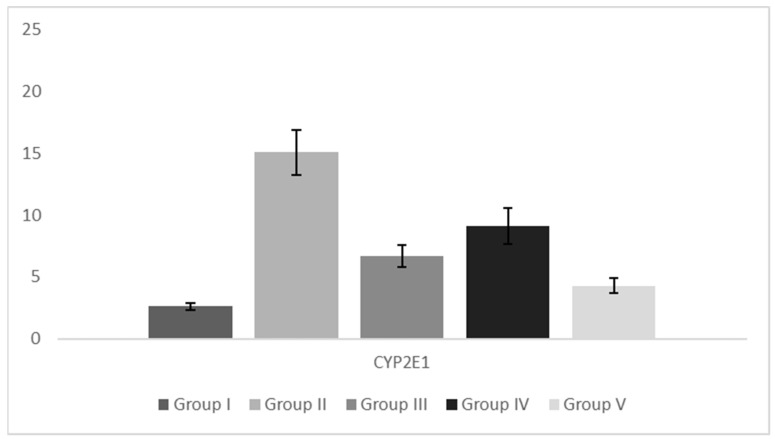
CYP2E1-positive liver tissue. Legend: Group I—animals treated with saline (1 mL/kg); Group II—hypercholesterolemic animals treated with saline (1 mL/kg); Group III—hypercholesterolemic animals treated with carob extract (400 mg/kg); Group IV—hypercholesterolemic animals treated with simvastatin (10 mg/kg); Group V—hypercholesterolemic animals treated with the combination of carob extract (400 mg/kg) and simvastatin (10 mg/kg).

**Figure 3:**
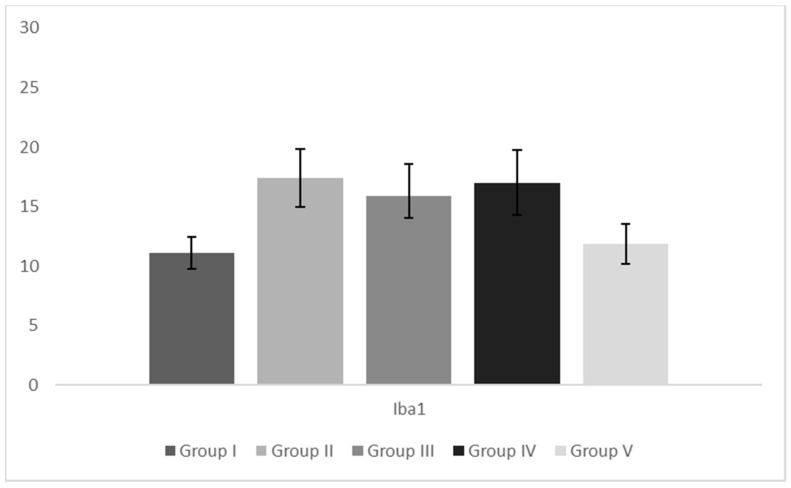
Ratio of Iba1-positive cells in liver tissue. Legend: Group I—animals treated with saline (1 mL/kg); Group II—hypercholesterolemic animals treated with saline (1 mL/kg); Group III—hypercholesterolemic animals treated with carob extracts (400 mg/kg); Group IV—hypercholesterolemic animals treated with simvastatin (10 mg/kg); Group V—hypercholesterolemic animals treated with the combination of carob extract (400 mg/kg) and simvastatin (10 mg/kg).

**Figure 4:**
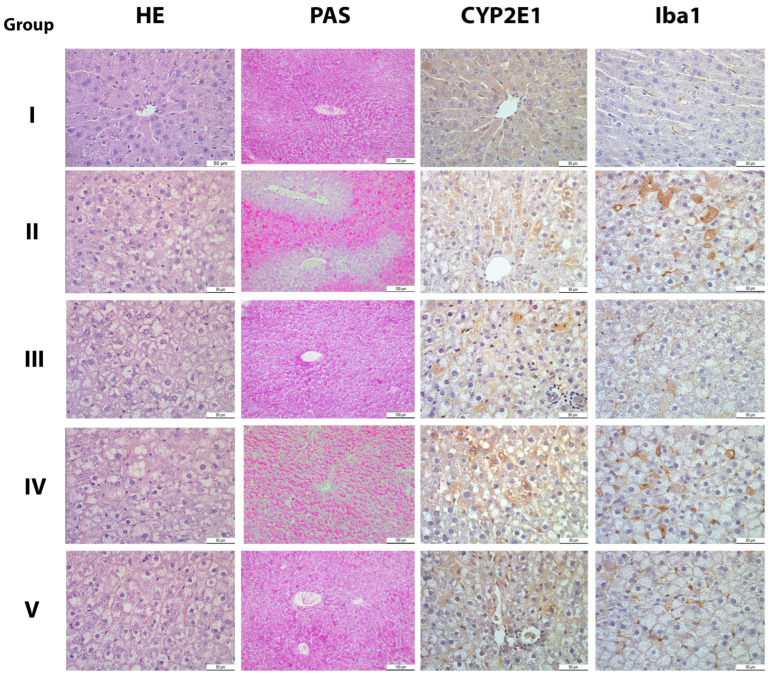
Histological and immunohistochemical analysis of liver tissue (40×, HE—hematoxylin and eosin; 20×, PAS—Periodic Acid-Schiff; 40×, anti-CYP450 monoclonal antibody, clone 2E1; 40×, anti-Iba1 monoclonal antibody). Legend: HE—(**I**) normal liver histology with low expression of CYP2E1 and Iba1; (**II**) HE—steatosis, PAS—depletion of glycogen, CYP2E1 and Iba1—increase in CYP2E1 positive hepatocytes and Iba1+ macrophages, with alteration in its shape; (**III**) HE—nearly annulled steatosis and ballooning, PAS—slightly reduced glycogen depots, CYP2E1 and Iba1—reduced CYP2E1 positive hepatocytes and Iba1+ macrophages; (**IV**) HE—alleviated hepatic tissue impairment (steatosis is present but less compared to Group II), PAS—slightly reduced glycogen depots, CYP2E1 and Iba1—reduced expression, persistent alteration in shape of macrophages; (**V**) HE—liver morphology similar to control, PAS—slight reduced glycogen depots; CYP2E1—significantly reduced expression, Iba1—reduced number of Iba1+ macrophages, with restoration of its usual shape. Group I—animals treated with saline (1 mL/kg); Group II—hypercholesterolemic animals treated with saline (1 mL/kg); Group III—hypercholesterolemic animals treated with carob extract (400 mg/kg); Group IV—hypercholesterolemic animals treated with simvastatin (10 mg/kg); Group V—hypercholesterolemic animals treated with the combination of carob extract (400 mg/kg) and simvastatin (10 mg/kg).

Surface of CYP2E1 positive liver tissue (Figure 2), as a result of increased CYP2E1 ac-tivity is much higher in hypercholesterolemic animals compared to controls in Group I, while the optimal reduction of CYP2E1 activity is achieved through cotreatment with both carob and simvastatin. Ratio of Iba1 positive cells in liver tissue showed slight differences among groups: compared to Groups I hypercholesterolemic animals have higher count of Iba1-positive cells, while cotreatment with both carob and simvastatin reduced Iba-positive cells to a level near Group I (Figure 3). CYP2E1 staining (Figure 4) in control (Group I) liver tissue was present in the form of weak staining in rare, solitary hepatocytes near terminal hepatic venules (centrilobular hepatocytes). In liver sections from hypercholesterolemic animals (Group II), CYP2E1 immunostaining increased in intensity (moderate to strong), and the pattern of lobular distribution was altered compared to Group I samples. CYP2E1-positive cells were scattered throughout the lobule, usually (but not exclusively) following the distribution of steatosis. The amount of CYP2E1-stained tissue was significantly higher in Group II compared to Group I (Figure 4). Such as reduction of hepatocyte damage, the carob/simvastatin regimen reduced CYP2E1 expression significantly compared to Group II and to a greater extent compared to carob or statin alone (Figure 2 and Figure 4).

Iba1-positive (Iba1+) cells were present in Group I liver tissue, but in Group II, the ratio of Iba1+ cells rose evidently/significantly (Figure 3 and Figure 4). Macrophages appeared larger and more rounded, with multivacuolated (foamy) cytoplasm. Compared to the hypercholesterolemic group treated with saline, the infiltration of macrophages was attenuated in Groups III and IV, while carob/simvastatin co-administration led to both a reduction in the ratio of Iba1+ cells and a restoration of macrophage size and shape (Figure 4).

## 4. Discussion

Carob is recognized for its anti-obesity, lipid-lowering, anti-oxidative, and anti-inflammatory effects, as previous studies have shown [15,16,17,18,19,29,31]. Our study confirmed some of these effects. Further, we evaluated the safety of the remedy both alone and in combination with simvastatin.

### 4.1. Effects on Obesity

The findings of the present study suggest that rats fed a high-fat diet and treated with carob pulp extract had significantly lower weight gain compared to the untreated rats. The possible mechanisms for this phenomenon are numerous and include the inhibition of adipocyte proliferation and differentiation [31], reduction of food intake [32], decline in body glucose and lipid content [36], increase in serum adiponectin [27,30], decrease in leptin resistance [33,37,38], and inhibition of fat accumulation in the liver [31]. Our study addresses two of these mechanisms.

Leptin levels in rats fed a high-fat diet were remarkably higher compared to the control group, indicating the development of resistance in leptin receptors to the hormone [37]. Introducing carob to the high-fat diet group resulted in a significant decrease in leptin levels, possibly indicating the recovery of leptin receptors and an enhanced leptin effect. However, this increase in the leptin effect could only partially explain the suppression of weight gain in our study. The initial effect of leptin is the satiety effect, which normally increases food intake [37]. Since food intake did not significantly differ between groups in our study, this mechanism alone cannot provide an explanation. The second effect of leptin is through the increase in energy expenditure [37], which could possibly explain the decline in weight gain observed. Additionally, leptin exhibits hypoglycemic and anti-hyperlipidemic properties [38], which may contribute to the overall effect.

Importantly, the combination of carob with simvastatin led to an even greater decrease in leptin levels. However, contrary to our expectations, the group treated with the combination of carob and simvastatin showed higher body weight gain compared to the group treated with carob alone. Therefore, since this is the first study, to our knowledge, to evaluate the effect of carob on leptin, further investigation is warranted to explore the hypothesized mechanism mentioned above.

Another possible mechanism of the weight-lowering effect is the inhibition of liver fat accumulation, as our findings suggest that the post-mortem liver weight was significantly lower in the groups treated with carob and/or simvastatin compared to the untreated high-fat diet-fed groups.

Several studies on animal models have reported the impact of carob on body weight and proposed various explanatory mechanisms [31,32,36]. Fujita et al. [31] showed that supplementation with carob pod polyphenols (CPPs) significantly decreased body weight gain in high-fat-fed mice. They attributed this effect to the CPPs’ ability to inhibit fat accumulation in the liver and suppress adipocytes’ hypertrophy and differentiation through post-transcriptional regulation of CCAAT/enhancer-binding protein beta (C/EBPβ). As explained above, this mechanism could also account for the decreased weight gain observed in our study. De la Fuente-Fernández et al. [32] revealed that supplementation with carob extract induced significant weight loss in high-fat diet-fed mice, primarily through its satiety effect, resulting in reduced food intake. Forestieri et al. [36] showed a suppressed weight-increase curve in high-fat diet-fed rats supplemented with carob gums. They attributed this effect to the hypoglycemic and anti-lipidemic properties of carob. Considering that leptin also has glucose- and lipid-lowering potential [38], this mechanism could provide an explanation for our findings as well.

In summary, the complex mechanisms underlying carob’s anti-obesity properties warrant further investigation. Nevertheless, it is indisputable that these effects exist.

### 4.2. Effects on Dyslipidemia

Our study revealed some of the carob’s properties in the regulation of dyslipidemia, but only in combination with simvastatin.

Regarding TC, LDL-C, and LDL/HDL ratio, a significant decrease in these indices compared to the hypercholesterolemic group treated with saline was found only in the groups treated with simvastatin or carob/simvastatin combined. Many studies showed the lipid-lowering effect of carob-only regimens [16,17,18,19,24,25,26,27,28]. The possible explanations why we did not prove this effect are the usage of different animals, the dosage of carob pulp, and the duration of the treatment.

In respect of HDL-C, the group treated with the combination of carob and simvastatin had remarkably higher HDL-C compared to the untreated group and the group treated with the carob-only or simvastatin-only regimen. It is a well-known fact that statins induce a rise in HDL-C [39]. Also, numerous studies showed that carob increases levels of HDL-C [16,17,18,19,29]. However, no study, to the best of our knowledge, investigated the combined effect of simvastatin and carob on HDL-C. The present study showed that this combination is superior to the single therapy with statin or carob. This finding encourages future research on animals and humans intending to implement carob as an add-on therapeutic agent to the standard statin protocols. Also, future studies should explore the complex mechanisms behind these effects. One of the possible explanations is the synergistic effect of simvastatin and carob on the liver. It is known that statins inhibit cholesteryl ester transfer protein (CETP), thereby inducing a rise in apolipoprotein A-I and HDL-C [40]. Carob, on the other hand, could possibly induce the rise of HDL-C by multiple already-mentioned lipid-lowering mechanisms. Additional mechanisms of the carob-induced rise of HDL-C explained by our study could be due to the observed decrease in leptin levels and anti-obesity effects. As leptin induces up-regulation of scavenger receptor type B1, thereby promoting clearance of HDL by the liver, decreased levels of leptin would cause higher plasma levels of HDL-C [41]. In obesity, levels of HDL-C are low due to increased leptin, increased CETP production, decreased adiponectin, increased HDL-C clearance by hepatic and endothelial lipase, and reduced cholesterol efflux to HDL-C in adipocytes [42]. As carob showed its anti-obesity effect, it possibly diminished these obesity-associated HDL-C-lowering factors, especially leptin levels, thereby inducing the HDL-C to rise.

### 4.3. Effects on Liver and Kidney Function Tests

Concerning the effects on ALT and AST, animals treated with carob had significantly lower levels of both liver enzymes compared to the control group and also showed decreased ALT levels compared to the high-fat-fed group treated with saline. This finding once again proved carob’s potential hepatoprotective activity [35]. Also, given the rare but possible statin-induced liver damage as a treatment complication, it would be reasonable to conduct further clinical trials intending to introduce the hepatoprotective agents along with routine statin therapy [43]. In respect of the renal function tests, urea was remarkably lower in animals treated with carob compared to the control group, whereas creatinine and uric acid did not differ significantly between the groups. These findings suggest that carob has no harmful effects on kidney function. What’s more, it may protect the kidney from damage, as other studies proved as well [44,45].

### 4.4. Effect on Histological and Immunohistochemical Features of Liver Tissue

Nonalcoholic fatty liver disease (NAFLD) is a chronic liver disease and a component of the metabolic syndrome. Histopathological changes of the liver vary from asymptomatic hepatic lipid accumulation/steatosis in non-alcoholic fatty liver (NAFL) to nonalcoholic steatohepatitis (NASH). As expected, the high-fat diet of animals in Group II leads to histopathological changes corresponding to non-alcoholic fatty livers. Beneficial effects of carob are reflected in the absence of steatosis and inflammation in the hyperlipidemic group with carob treatment and strikingly reduced steatosis in carob/simvastatin treatment. In our study, sole carob had a more potent antisteatotic effect compared to simvastatin alone. Excess lipid accumulation in hepatocytes leads to hepatocyte injury and hepatic inflammation, among others. Macrophages (Kupffer’s cells) are the main population of inflammatory cells in the liver. Inflammatory signals from hepatocytes lead to macrophage activation, which further secretes several pro-inflammatory cytokines, leading to more severe liver damage associated with the pathogenesis of NAFLD/NASH [46]. Iba1 is a marker of macrophages, and in Group II, the ratio of Iba+ macrophages increased significantly. This is in accordance with previous research by Yoshii et al., who determined that the number of Iba1+ macrophages increases with an increase in cholesterol content in the diet [46]. It is known that upon activation, macrophages can take one of two phenotypic profiles, M1 or M2. M2 are producing anti-inflammatory cytokines, but uptake of lipids by resident liver macrophages alters their profile to that of M1. Also, monocytes derived from peripheral blood, upon arrival in liver tissue, take the M1 pro-inflammatory phenotype. M1 macrophages secrete pro-inflammatory cytokines and cause further hepatocyte steatosis [47]. There is an obvious decrease in the percentage of Iba1+ cells after carob and carob/statin administration. It may be an effect of the antidyslipidemic, weight-lowering, and leptin-lowering properties of the tested substances. Damage to hepatocytes is decreasing, so there is no need for an influx of monocyte-derived macrophages. However, we must address the fact that the detected differences in the Iba1+ cell ratio are not statistically significant compared to the hyperlipidemic group. An explanation may be found in the interplay of M1/M2 phenotype shifting. It is reported that both M1 and M2 may be Iba1-positive. It is possible that upon carob administration, its lipid-lowering effects impact M1 to M2 alteration, so the present macrophages downregulate the inflammatory response and facilitate tissue repair. It should be mentioned that, although M1/M2 terminology is generally accepted, there are many uncertainties in regard to the mechanisms of M1 activation as well as the subclassification of M2 macrophages [47]. This should be further investigated in terms of carob effects on macrophage activation. We demonstrated that along with changes in the number of Iba1+ cells, there is a significant impact on their cellular morphology/shape.

Oxidative stress has been suggested to play a role in the pathogenesis of NAFLD. Sources of oxidative stress include cytochrome P450 2E1 (CYP2E1), lipid peroxidation, mitochondrial dysfunction, cytokine induction, NADPH oxidase, etc. [48]. In our study, CYP2E1 expression in the liver rose in Group II compared to Group I. This finding corroborates other studies, saying that CYP2E1 was increased in obesity, fatty liver, NAFLD, and NASH in both humans and rodents, and this increase appears to correlate well with the severity of NAFLD [7,10,11,12].

The pathogenesis of NAFLD is complex, and the theory of “two hits” is well known [7]. The first hit is steatosis, caused mainly by IR, while the second hit is liver damage and inflammation caused by oxidative stress (OS). Notably, OS is thought to be involved in the entire development of NAFLD and is known to be induced by excessive reactive oxygen species (ROS) [49]. CYP2E1 can oxidize a variety of small-molecule substrates, including xenobiotics, ethanol, and fatty acids, producing superoxide anion, a very potent reactive oxygen species (ROS), which can serve as part of the second hit to advance the severity of NAFLD [48]. Thus, for the treatment of NAFLD, it is of great interest to establish whether carob has the potential to affect CYP2E1 expression/activation. According to our results, the high-fat-fed control group had significantly higher CYP2E1 expression compared to group I. This group had significant weight gain, so it is all in accordance with other studies showing that high-fat diets and obesity lead to CYP2E1 overexpression [50]. Carob alone or in co-administration with simvastatin induced a significant decrease in CYP2E1 expression, which strongly suggests its protective role in hepatocyte damage and the development and progression of NAFLD/NASH. This beneficial effect may be induced by the anti-dyslipidemic or anti-obesity properties shown in this study, as well as the antioxidative properties of carob, shown in previous studies on the paracetamol-induced hepatotoxicity model in mice [35].

## 5. Conclusions

This study confirmed the anti-dyslipidemic, anti-obesity, and hepatoprotective potential of carob pulp alone or in combination with simvastatin in the treatment of high-fat diet-fed rats. The impact of carob on lipid profile has been shown only when combined with simvastatin, with emphasis on the increase of high-density lipoprotein cholesterol, possibly through weight-lowering and leptin-reducing properties. The carob-only regimen showed a remarkable impact on decreasing weight gain, along with a significant decline in serum leptin levels and liver mass. The hepatoprotective effect of carob was also proven, in addition to its safety regarding liver and kidney function. Although the ameliorative potential of this plant-based remedy is indisputable, it is unsure whether carob alone could be effective in the management of dyslipidemia and obesity. Therefore, healthy lifestyle habits, statins, and anti-obesity drugs should remain the backbone of standard treatment regimens. Having all this in mind, it would be reasonable to conduct further larger-scale animal studies and human clinical trials in order to implement carob as an add-on therapeutic option for the treatment of dyslipidemia and obesity.

## Figures and Tables

**Table 1:** Experimental animal groups and their characteristics.

Group Order	Group Name	Treatment Received	Numbers of Animals	Treatment Duration
Group I	Control group	saline, 1 mL/kg (p.o.)	8	4 weeks
Group II	Hypercholesterolemic animals treated with saline	saline, 1 mL/kg (p.o.)	8	4 weeks
Group III	Hypercholesterolemic animals treated with carob extract	carob, 400 mg/kg (p.o.)	8	4 weeks
Group IV	Hypercholesterolemic animals treated with simvastatin	simvastatin, 10 mg/kg	8	4 weeks
Group V	Hypercholesterolemic animals treated with the combination of carob extract and simvastatin	carob, 400 mg/kg (p.o.) + simvastatin, 10 mg/kg (p.o.)	8	4 weeks

**Table 2:** Initial and final body weight, weight gain, liver weight, and food and water intake according to the animal groups.

Variable	Group I	Group II	Group III	Group IV	Group V
Initial body weight (g)	214.4 ± 23.7	257.8 ± 19.4	250.2 ± 15.2	207.8 ± 35.6	235.6 ± 17.6
Final body weight (g)	366.2 ± 37.3	452.2 ± 11.3	405.4 ± 32.1	390.4 ± 37.7	413.8 ± 27.7
Weight gain (g)	151.8	194.4 *,^§^	155.2 ^†^	182.6 *^,§^	178.2 *^,§^
Liver weight (g)	11.20 ± 1.98	21.16 ± 2.38 *^,§^	16.36 ± 1.57 *^,†^	17.19 ± 2.24 *^,†^	16.77 ± 2.12 *^,†^
Food intake, g/24 h/animal	28.69 ± 1.78	21.75 ± 2.13 *	20.94 ± 1.87 *	21.18 ± 1.96 *	22.10 ± 2.08 *
Water intake, ml/24 h/animal	53.74 ± 2.57	60.77 ± 3.34 *^,§^	70.40 ± 3.03 *	62.75 ± 2.64 *^,§^	62.30 ± 2.85 *^,§^

Legend: Data are presented as mean ± SD; * *p* < 0.05 comparing to Group I; ^†^ *p* < 0.05 comparing to Group II; ^§^ *p* < 0.05 comparing to Group III. Group I—animals treated with saline (1 mL/kg); Group II—hypercholesterolemic animals treated with saline (1 mL/kg); Group III—hypercholesterolemic animals treated with carob extract (400 mg/kg); Group IV—hypercholesterolemic animals treated with simvastatin (10 mg/kg); Group V—hypercholesterolemic animals treated with the combination of carob extract (400 mg/kg) and simvastatin (10 mg/kg).

**Table 3:** Lipid profiles in different animal groups.

Variable	Group I	Group II	Group III	Group IV	Group V
Triglycerides (mmol/L)	1.23 ± 0.42	0.99 ± 0.55	1.02 ± 0.26	0.82 ± 0.20	0.94 ± 0.40
Total cholesterol (mmol/L)	1.90 ± 0.11	3.93 ± 0.59 *	3.21 ± 0.39 *	2.55 ± 0.48 *^,†^	2.44 ± 0.40 *^,†^
HDL cholesterol (mmol/L)	0.74 ± 0.15	0.49 ± 0.11 *	0.61 ± 0.18	0.66 ± 0.17	0.71 ± 0.10 ^†^
LDL cholesterol (mmol/L)	0.62 ± 0.28	3.45 ± 0.48 *	2.79 ± 0.41 *	2.03 ± 1.04 *^,†^	1.86 ± 0.99 *^,†^
LDL/HDL ratio	0.83 ± 0.38	5.15 ± 0.83 *	4.36 ± 0.95 *	2.73 ± 1.63 *^,†^	2.06 ± 0.46 *^,†^

Legend: Data are presented as mean ± SD; * *p* < 0.05 comparing to Group I; ^†^ *p* < 0.05 comparing to Group II. Group I—animals treated with saline (1 mL/kg); Group II—hypercholesterolemic animals treated with saline (1 mL/kg); Group III—hypercholesterolemic animals treated with carob extract (400 mg/kg); Group IV—hypercholesterolemic animals treated with simvastatin (10 mg/kg); Group V—hypercholesterolemic animals treated with the combination of carob extract (400 mg/kg) and simvastatin (10 mg/kg).

**Table 4:** Liver function tests and renal function tests according to the animal groups.

Variable	Group I	Group II	Group III	Group IV	Group V
AST (U/I)	98.80 ± 6.72	91.00 ± 6.58	83.17 ± 9.70 *	93.22 ± 13.71	92.14 ± 13.46
ALT (U/I)	46.0 ± 6.82	45.90 ± 7.07	32.71 ± 6.40 *^,†^	47.10 ± 7.72 ^§^	43.71 ± 7.34
Total bilirubin (µmol/L)	1.65 ± 0.15	1.69 ± 0.17	1.63 ± 0.10	1.82 ± 0.11	1.68 ± 0.12
Urea (mmol/L)	7.22 ± 0.50	5.54 ± 0.61 *	4.88 ± 0.54 *	5.41 ± 0.77 *	5.34 ± 0.47 *
Creatinine (μmol/L)	42.92 ± 3.19	46.24 ± 2.04	47.60 ± 3.11	47.91 ± 1.62	48.41 ± 2.87
Uric acid (μmol/L)	7.00 ± 1.0	6.71 ± 0.95	7.43 ± 1.51	7.00 ± 1.32	7.75 ± 1.39

Legend: Data are presented as mean ± SD; * *p* < 0.05 comparing to Group I; ^†^ *p* < 0.05 comparing to Group II; ^§^ *p* < 0.05 comparing to Group III. Group I—animals treated with saline (1 mL/kg); Group II—hypercholesterolemic animals treated with saline (1 mL/kg); Group III—hypercholesterolemic animals treated with carob extract (400 mg/kg); Group IV—hypercholesterolemic animals treated with simvastatin (10 mg/kg); Group V—hypercholesterolemic animals treated with the combination of carob extract (400 mg/kg) and simvastatin (10 mg/kg).

## Data Availability

The data presented in this study is contained within the article.
